# Diagnosis

**Published:** 1892-09

**Authors:** J. R. Bell

**Affiliations:** Cleveland, O.


### Diagnosis.

BY J. R. BELL, D.D.S., CLEVELAND, O.

Read before the Northern Ohio Dental Society, Cleveland, May, 1892.

There are things which we do daily that we know why and how to do, which if we were called upon for an explanation, would find ourselves deficient in language to express, and this, gentlemen, is the feeling we have in imparting our ideas upon the subject of Diagnosis.

We have heard of men, of whom it is said, "could tell all and even more than they knew; and others, who knew more than they could tell" we beg to be classed with the latter in this one instance, at least.

Diagnosis, the art or science of signs or symptoms, by which one disease is distinguished from another. The definition covers such a scope, even in the abnormal conditions with which we have to deal, that we make no effort to surround it, but only present our art of discerning symptoms of various affections most common in our specialty. There are stages of symptoms in and remote from the organs with which we have to deal, which make it (until the disease develops) impossible to safely pronounce the same, therefore, a reserved opinion is advisable, but as it is how we distinguish and not what we should or should not do with a case that we are to talk, we proceed; but first allow us to take advantage of the opportunity to say, that correct diagnosis is the foundation principle upon which success depends. Then our pathology, sanitary laws, hygiene, etc., are merely accompaniments to this. Then our intuitive vision, which is capable of knowing without education or reasoning, fits us for action, but exclude judgment and education, and man can act only mechanically or by comparison. Now, let us talk about some of the important affections indicated in the irritation of first dentition, together with inflammation, disposition disturbed, rise of temperature, thirst, and if relief is not afforded, diarrhœa, and convulsions. In abnormal dentition the same symptoms, with more tense, shining gums, more fever with skin eruptions.

*Convulsions of Dentition.*—Premonitory symptoms are changeable temper, unusual luster of the eyes, indifference to take food, twitching of facial muscles, biting of teeth, when paroxysms are indicated the face will turn red or purple, stiffening of the body, respiration suspended, unconsciousness, protrusion of tongue, rapid intermittent pulse.

*Inflammation of the peridental membrane* is demonstrated by uneasiness and fullness, at first relief is experienced by occlusion of teeth, chronic form pressure causes dull pain, tooth is raised in its socket and meets the opposing teeth in advance of all others, slightly loosened, indicating plainly a thickening membrane, the gum margin is a high color, but there are often exceptions to this rule, in tissue more easily broken down from diathesis, more of course will be found involved in proportion to diathesis affection, time, etc. In subjects of this nature we learn to anticipate, if inflammation does not yield to treatment, either ulceration or alveolar abscess.

In modified symptoms of this affection, intermittent pain, tooth gradually becomes less of a fixture, indicating detachment of peridental membrane by engorged and broken down blood vessels. Continued symptoms indicate either local or systemic disturbances. If allowed to progress, devitalization of pulp will naturally occur.

To locate peridental inflammation on teeth of one or more roots, gentle pressure applied at different angles will usually cause slight pain and show the disease to be upon the side from which the pressure is produced.

*Aveolar Abscess.*—At first patient will explain having had pain constantly. Then increased by each pulsation, from a varied length of time, say two or three days, a sense of swelling over diseased root, later if the tissues break down more rapidly than the system absorbs the pus and carries it off, there will be a pointing and discharge, at which time the above symptoms become less pronounced, and are soon unnoticed. During the development and until relief is obtained, there are usually constitutional symptoms, offensive breath, rise in temperature, thirst, headache, and foul tongue. Throbbing pain denotes pus formation.

*In chronic form.*—The above symptoms become less pronounced and the discharge of a small quantity of pus, either coming from the mouth of the fistula or it can be made to escape by pressure upon the neck of the sinus.

To ascertain the origin of an elastic fluctuating swelling on any part of the face or neck, draw the fingers gently to, and from the terminus of the swelling and the tooth indicating the cause, when pain will be produced by strangulation of blood and pressure upon nerves through the vessels associated, and if there is a blind abscess the desired effect will be obtained by changing position of disorganized blood corpuscles.

The indications of an abscess threatening to point externally, are understood by red, shining, distended skin, thin and scaly surface from which epidermis may be peeled off.

*Abscess or disease of antrum* is considered the most difficult to diagnose of any disease with which we have to deal; this is true only in imagination, for when the anatomy of the face and the frequent communication of the roots of the teeth and this cavity are understood, together with the symptoms that follow with variation of course, the diagnosis is simple.

The quantity of pus and pain is intermittent, according to the normal or depleted state of the general system. Constitutional disturbance is one of the strongest proofs, and if not visible should be inquired into. Cheek will be found hot, slightly swollen, with frequent sharp pains which increase in proportion to above symptoms, until the pain becomes of a throbbing character and may produce rigors and fever peculiar to suppuration. The molar teeth on the affected side described as elongated from depression, which may prevent proper occlusion, the palate instead of being concave becomes convex. The nostril becomes encroached upon, and the floor of the orbit sometimes pushed up in such a way as to partially force the eye from its socket, viz., in protracted cases, and the eyesight may become affected by stretching the optic nerve; about this stage of the disease either the effects of "blood poisoning" will appear, or some sinus discovered through which the pus can escape, either into the nose, through the floor of the antrum, around the necks of the teeth, or it may be found first discharging into the eye, or through the cheek. When if the fistula is large enough to afford free escape of the pus, the pain will cease until the cavity fills again.

*Alveolar ulceration* may be distinguished by purulent matter oozing out around the necks of the affected tooth or teeth, or through the gum. In regular abscess new tissue is constantly being formed, while in ulceration normal tissue is continually being disorganized. We will find a watery discharge, translucent, and oftentimes odorless, while in case of an abscess, the discharge is opaque and generally offensive. We find too little pain or swelling in ulceration; this is the most dangerous form of disease with which we have to do, because of the possibility of the ulcer becoming malignant, and the liability of its poisoning the operator or his succeeding patients for want of thorough antiseptic condition of both hands and instruments.

In its chronic form we know that the alveolus and pericementum adjacent to the disease is being slowly carried off, and we may naturallv expect calcareous deposits upon the roots of teeth not yielding kindly to treatment.

*Necrosed Alveolus* is clearly understood when the gum is dark purple and the discharge is offensive, gums soft, bleed at the slightest touch, and appear loose from the bone beneath.

*Mercurial Salivation.*—In a mild form we find simply a red margin of the gum, which very soon, however, becomes soft and tender to pressure; then, too, there is always the metallic taste described and the fetor of the breath. In more marked cases of poisoning, the saliva becomes profuse and the patient has difficulty in controlling it. The metallic taste, soreness, stiffness of the jaws, increase in proportion. In this stage sloughing of the gums will commence.

*Syphilitic Ulcers.*—The whole gum surface is red and swollen, with ulcerated margins often exposing the necks of the teeth, although they may not be found loosened as in other inflammatory conditions there is usually a bloody discharge from the gum margins. We should readily understand these symptoms, because of the danger of contagion and transmission.

*Scrofula.*—We find it more marked in the young subjects, and although there are exceptions, there is always a tendency, and it is a pretty sure sign of scrofulous diathesis, where the lymphatic glands are found enlarged; this sign, together with more or less skin eruptions, are unmistakable characteristics of scrofula.

*Tumor of the upper jaw* is generally indicated by a gradually increasing prominence of the cheek, many times involves nostril and mouth and closes the eye.

*A malignant tumor of the jaws* is almost invariably of rapid growth, which is hard, invades surrounding tissues with a tendency to fungate like mushrooms.

*There are two kinds of Cysts, called Dentigerous.*—This word is derived from the two Latin words, "dens," a tooth, and "gero," to carry, to bear. First, those found upon the roots of well developed teeth; second, those more frequently connected with unerupted or imperfectly developed teeth, and they are common to either jaw. There is disfigurement, a sense of weight and contraction of affected parts, frequently constitutional disturbance.

HEATH: in speaking of the clinical history of cysts connected with the teeth, describes them as painless, expansion of the alveolus of either jaw, but more frequently of the upper, with crackling of the bone on pressure and the ultimate absorption of the same.

In the one feature of his diagnosis we differ, viz., in the absence of pain. A cyst, he says, when distended presents a bluish appearance, and when very large gives distinct fluctuation.

*An unerupted* or impacted tooth is indicated by a hard tumor like growth over the alveolar ridge, frequently, if on the upper jaw, extending along the plane of the palate bone, on the surface of the maxilla, and in the shape of corresponding tooth.

*Necrosis, or death of jaw bone*, is similar in its first stages to peridontitis, but later there is a vast difference; we find the gum thickened; tumid and very red, with pus oozing from the borders around the teeth, with recession of the same.

*Hypertrophy of the gums* is found mostly among two classes, who are extremes in habit; one, seldom if ever brush or pick their teeth; the other, clean theirs and produce the above affection through ignorance, by the use of tooth-powder, advised and sold to the victim by dentist or druggist.

"Where there's a penny to be gained, its unwise to truthfully advise."

Pardon our divergence from the subject. The temptation was too great for us to refrain from expressing our indignation for this injurious practice of advising powder as a dentifrice.

We distinguish hypertrophy readily. The gums are of a dark color, and increase so as to partially or fully cover the teeth ; accompanying these symptoms are disagreeable breath and increase of saliva.

*Alveolar Pyorrhœa.*—The symptoms approximate those of syphilis, mercurial salivation or hypertrophy ; it is distinguished from other affections by extreme sensitiveness of gums, which have either a polished glossy-like surface, or knotty and rough. In simple form there is merely a congested appearance of the gum, but in its chronic state teeth are loose from gum margins, in which pockets are formed and a wasting away of the alveolar processes ; later the only attachment is a tough ligamentus connection from a portion of the root, which seems to strengthen by use of the tooth, but later gives way altogether unless local systemic and preventive means are adopted.

*Irritation of the Dental Pulp.*—Like general nervous irritation, we must look to causes arising from organs affected by other disease, and so on through a chain of troubles, very remote oftentimes from the one in which the greatest pain has finally settled.

If the general health is impaired through sickness, excessive sedentary labor, lack of nutrition, gestation, lactation, anæmic or climatic condition, the cause and effect should be noted and our actions governed accordingly.

Sex, age, temperament, diathesis and habit, separately and collectively, demand careful but necessarily only a passing glance to determine the possibilities in cases of this and other similar affections as they come under our care. In simple irritation the first sensation is consciousness or an unnatural feeling in some of the teeth upon the affected side, the person himself and we too are puzzled to locate it carefully at first, as the pain becomes of a boring or gnawing character, disturbing mastication and rest, it becomes acute, then the separate organ is singled out, but neighboring teeth will also be disturbed sympathetically. When this stage arrives the pain in the tooth becomes very distinct, others are unnoticed, except at intervals when there will be a remission, then it may recur instantly, and especially when the person assumes a recumbent position. We are doubtful if the pulp can be prevented, indefinitely, from devitalization when this stage is reached.

*Chronic inflammation of the dental pulp* is distinguished from irritation and acute form, because the pain is more wandering, like neuralgia, and is excited by changes of temperature, especially from dry to wet, and is also of an intermittent character.

*A fungous growth on the pulp* is like a small vascular tumor, but there are exceptions, when it may be found as large as a navy bean. Its diagnosis is of little consequence as the entire pulp has to be destroyed, and usually that, together with the filling of the tooth, is simple.

*Diagnosis of ossification of the pulp* is difficult, but is important, and should be distinguished from other affections producing symptoms nearly in common, because different means are necessary to accomplish the desired end. The pain is like that of a wound healing by first intention, there is commonly a gnawing sensation, causing consciousness that some tooth on the affected side is not right. It may affect the side of the head or face, but severe pain is not constant. The affected tooth remains natural in color and free from soreness for days and even weeks in some instances, until it is pronounced a nuisance. Again we find partial ossification in carious teeth in which there has been no disturbance.

We find ossification of the pulp in the youth of old age, viz., in the neighborhood of the fiftieth year, when from abrasion of the teeth and ossification of the pulp they either require crowns or gold screws and tips, then it is necessary to determine their vitality. Heat a blunt instrument to a degree just endurable upon the finger nail, now quickly apply to the tooth in question, when there will be a response by a demonstration of consciousness or complete insensibility.

*Dentine abnormally sensitive*, or so much as to require treatment preparatory to permanent restoration, is indicated by general debility of the system, white chalky or leathery disintegration, susceptible to thermal change, vitiated saliva, showing acid reaction, gum margins irritated from white pasty deposit, want of use, but showing absence of care and nourishment.

*Abrasion of the teeth* is first indicated by a polished, glassy-like occluding surface, in the more advanced stage the crown of the tooth becomes concave, showing the form of the pulp-chamber, which is itself partially calcified.

*In dental exostosis* the symptoms are very like those we meet with in ossification of the pulp, continued, however, the character of the trouble is distinguished, because of the prominence on the plane of the alveolar ridge.

*Defects of Tooth Structure.*—Anomalies of this character were formerly denominated atrophy, but it does not apply, from the fact that these defects so often seen are from faulty formation of structure and not wasting after eruption. In examination of these defects their character, progression, and the age, and the teeth in which they are found, suggest not only methods for restoration, but the time the disturbance was present. For instance, the spots, grooves or pits, in any class of teeth, denote lack of proper nourishment through disease one or two years prior to its eruption. Example: Six year molar four to five years; incisors, sixth or seventh, and so on.

*In syphilitic teeth* the cutting edges are notched, cuspids are like pegs usually dark color and soft consistence.

Mr. Milner Fothergill says: "There is but little doubt but that the configuration of the teeth in gout has a distinct value." The peculiar characteristics are always pronounced. The teeth are short and thick, with heavy shoulders upon the lingual side, with encircling grooves, which give the teeth a step-like appearance. The gums are light and thin and retracted. The center of the tooth is dark color.

Many cases of atrophied teeth are the result of severe whooping cough. The enamel will be found soft and the dentine oftentimes greenish color.

*Puberty affect*, while not a disease, retards the growth and quality of the unerupted teeth, third molars suffer.

Nasal catarrh of our climate tends to produce, defects in the development of the teeth. White spots are signs by which we distinguish this class.

*Measles or Measles tooth* are teeth usually centrals with exfoliation of tooth germs, eroded edges, vertical grooved, teeth are thin and narrow.

*The Malaria Tooth.*—If this term is allowed, is soft, rough, with spongy gums inclined to inflammation.

*Rheumatic teeth* are hard, flinty, checked edges, yellow and firmly planted.

*Scrofula.*—The teeth in subjects so affected have soft enamel, pinkish, muddy, opaque color, difficult to diagnose them from syphilitic teeth.

